# Long-term cardiovascular prognosis of patients with type 1 diabetes after myocardial infarction

**DOI:** 10.1186/s12933-022-01608-3

**Published:** 2022-09-06

**Authors:** Anne M. Kerola, Anne Grete Semb, Markus Juonala, Antti Palomäki, Päivi Rautava, Ville Kytö

**Affiliations:** 1 Inflammation Center, Rheumatology, Helsinki University Hospital, Helsinki, Finland; 2 Faculty of Medicine, University of Helsinki, Helsinki, Finland; 3 Preventive Cardio-Rheuma Clinic, Division of Rheumatology and Research, Diakonhjemmet Hospital, Oslo, Norway; 4 Department of Medicine, University of Turku, Turku, Finland; 5 Centre for Rheumatology and Clinical Immunology, Division of Medicine, Turku University Hospital, Turku, Finland; 6 Department of Public Health, University of Turku, Turku, Finland; 7 Turku Clinical Research Center, Turku University Hospital, Turku, Finland; 8 Heart Center, Turku University Hospital and University of Turku, Turku, Finland; 9 Administrative Center, Hospital District of Southwest Finland, Turku, Finland; 10 Department of Public Health, Faculty of Medicine, University of Helsinki, Helsinki, Finland

**Keywords:** Myocardial infarction, Type 1 diabetes mellitus, Epidemiology

## Abstract

**Background:**

To explore long-term cardiovascular prognosis after myocardial infarction (MI) among patients with type 1 diabetes.

**Methods:**

Patients with type 1 diabetes surviving 90 days after MI (n = 1508; 60% male, mean age = 62.1 years) or without any type of diabetes (n = 62,785) in Finland during 2005–2018 were retrospectively studied using multiple national registries. The primary outcome of interest was a combined major adverse cardiovascular event (MACE; cardiovascular death, recurrent MI, ischemic stroke, or heart failure hospitalization) studied with a competing risk Fine-Gray analyses. Median follow-up was 3.9 years (maximum 12 years). Differences between groups were balanced by multivariable adjustments and propensity score matching (n = 1401 patient pairs).

**Results:**

Cumulative incidence of MACE after MI was higher in patients with type 1 diabetes (67.6%) compared to propensity score-matched patients without diabetes (46.0%) (sub-distribution hazard ratio [sHR]: 1.94; 95% confidence interval [CI]: 1.74–2.17; p < 0.0001). Probabilities of cardiovascular death (sHR 1.81; p < 0.0001), recurrent MI (sHR 1.91; p < 0.0001), ischemic stroke (sHR 1.50; p = 0.0003), and heart failure hospitalization (sHR 1.98; p < 0.0001) were higher in patients with type 1 diabetes. Incidence of MACE was higher in diabetes patients than in controls in subgroups of men and women, patients aged < 60 and ≥ 60 years, revascularized and non-revascularized patients, and patients with and without atrial fibrillation, heart failure, or malignancy.

**Conclusions:**

Patients with type 1 diabetes have notably poorer long-term cardiovascular prognosis after an MI compared to patients without diabetes. These results underline the importance of effective secondary prevention after MI in patients with type 1 diabetes.

**Supplementary Information:**

The online version contains supplementary material available at 10.1186/s12933-022-01608-3.

## Background

Patients with type 1 diabetes mellitus are at a multifold risk for cardiovascular disease (CVD), and their first CVD events occur 10–15 years earlier than in the general population [1]. CVD is the leading cause of death in patients with type 1 diabetes and results in premature mortality in this high-risk population [2]. The substantially elevated risk for myocardial infarction (MI) in patients with type 1 diabetes has been acknowledged for decades and is steeply associated with levels of traditional CVD risk factors and genetic factors [3–5].

Identifying patient populations at a higher risk for CVD recurrence may aid in optimizing secondary prevention and motivating patients to strive for better CVD risk factor control. Several studies have demonstrated poorer outcomes after MI among patients with diabetes compared to those without, including higher mortality rates, risk of recurrent MI, and development of heart failure [6–8]. Most of these data, however, derive from cohorts with type 2 diabetes, diabetes of unspecified type, or, rarely, insulin-treated versus non-insulin-treated diabetes, whereas information on type 1 diabetes is notably lacking [6, 9–11]. Our recent study showed that patients with type 1 diabetes had higher 30-day and 1-year case-fatality rates after MI compared to patients without diabetes but with otherwise similar baseline features [12]. However, to our knowledge, no studies have explored long-term cardiovascular outcomes after MI specifically among patients with type 1 diabetes.

## Methods

### Aim

The purpose of this nationwide registry study was to explore the long-term cardiovascular prognosis of MI, including the risk of cardiovascular death, recurrent MI, ischemic stroke, and heart failure in patients with type 1 diabetes compared to controls without diabetes.

### Study design

The study data were retrieved from various national registries in Finland that cover the entire Finnish population. From the Care Register for Health Care in Finland (CRHC), we collected data on all MI patients aged ≥ 18 years admitted to MI-treating hospitals in Finland (n = 20, including five hospitals with emergency cardiac surgery) between January 1, 2005 and June 30, 2018. The index MI was identified with the ICD-10 code I21 as the primary diagnosis of hospital discharge. Only first-time admissions for MI to medical (including cardiology), surgical (including cardiac surgery), or intensive care wards during the study period were included. Exclusion criteria were death within 90 days after MI, admission duration > 90 days, and missing follow-up data (Additional file 1: Fig. S1). Hospital and ward transfers were treated as a single admission.

Patients were identified as having type 1 diabetes if they fulfilled the following four criteria: ICD-10 code E10 for type 1 diabetes in the CRHC at least once, an entitlement to special reimbursement for antidiabetic medication expenses, at least one insulin purchase, and no oral antidiabetic medication purchases within one year prior to the index MI (Additional file 1: Fig. S1). Special reimbursements for antidiabetic medications can only be granted if a physician writes a medical certificate that describes the rationale for the diagnosis of diabetes. A temporary need for insulin during pregnancy does not entitle a patient to special reimbursements for antidiabetic medication expenses. In Finland, antidiabetic medications are only available with a prescription from a pharmacy, and all antidiabetic medication purchases are collected in a national drug purchase database.

Controls without diabetes were identified as MI patients without records of any diabetes diagnosis in the CRHC (ICD-10 codes E10, E11, E12, E13, or E14), no entitlement to special reimbursement for antidiabetic medications, and no purchases of antidiabetic medications (including insulin) within one year prior to MI. Multivariable regression modeling and propensity score matching were used in the comparison of the type 1 diabetes and control groups. Subgroups analyses of the original cohort were performed in men and women, patients aged < 60 and ≥ 60 years, revascularized and non-revascularized patients, patients with and without atrial fibrillation, patients with and without heart failure, and patients with and without malignancy.

The primary outcome was composite major adverse cardiovascular event (MACE; cardiovascular death, or hospitalization for recurrent MI, ischemic stroke, or heart failure). Secondary outcomes were cardiovascular death, hospitalization for recurrent MI, ischemic stroke, or heart failure. Outcomes are defined in more detail in the Additional file 1: Methods section. Follow-up began 90 days after the index MI and continued up to 12 years. Follow-up data was available until December 31, 2018. In addition, we studied the usage of evidence-based secondary preventive cardiovascular medications within 90 days of discharge from the index MI. Comorbidities, ST-elevation MI, and medications were identified based on ICD-10 codes and operational codes in the CRHC, entitlements to special reimbursements for medication expenses, and Anatomical Therapeutic Classification codes in the registers of the Social Insurance Institution of Finland, as described previously [13, 14].

### Permissions for the use of registry data

Provided by the Finnish Institute for Health and Welfare, data on all hospital and emergency room admissions and major interventional procedures in Finland and data on malignancies were obtained from the CRHC registry and the Finnish Cancer Registry, respectively (permission no: THL/164/14.02.00/2021). Statistics Finland provided data on mortality and causes of death (permission no: TK-53-484-20). Data on entitlements to special reimbursements of medication expenses and prescription medication purchases were obtained from the Findata/Social Insurance Institution of Finland (THL/164/14.02.00/2021). As these registry data are routinely recorded and mandated by law, informed consent was not required, nor were the participants contacted. Legal grounds for the data handling are public interest and scientific research (EU General Data Protection Regulation 2016/679 (GDPR), Article 6(1)(e) and Article 9(2)(j); Data Protection Act, "Aim" and "Permissions for the use of registry data" sections).

### Statistical analysis

Differences between study groups were analyzed with t- and chi-squared tests (non-matched groups) or with paired t-test and McNemar’s test (matched groups). The effect sizes in the baseline characteristics between groups were evaluated by standardized mean differences (SMD). Time-dependent outcomes were studied using the cumulative incidence function and Fine-Gray regression to account for the competing risk of non-endpoint specific death [15]. Median follow-up was 3.9 years.

Logistic regression was used for the analysis of binary outcomes (medication usage). Propensity score was created with logistic regression and included sex, age, all co-morbidities and medications listed in Table 1, revascularization by percutaneous coronary intervention (PCI) or coronary artery bypass (CABG), ST-elevation, calendar year of index MI, and treating hospital (university versus non-university). For analysis of post-MI medication usage, propensity scoring was performed without medication usage. Variables were selected based on clinical experience.

**Table 1:** Baseline features of patients with myocardial infarction with type 1 diabetes or without any type of diabetes

	All patients				Matched patients			
	Type 1 diabetes	No diabetes			Type 1 diabetes	No diabetes		
Variable	n = 1508	n = 62,785	P value	|SMD|	n = 1401	n = 1401	P value	|SMD|
Age, years (SD)	61.2 (12.5)	68.7 (12.8)	< 0.0001	0.587	61.8 (12.6)	62.2 (12.9)	0.255	0.035
Men	59.8%	65.4%	< 0.0001	0.117	60.1%	59.7%	0.803	0.009
Co-morbidities								
Alcohol abuse	4.8%	3.1%	0.0004	0.084	4.7%	4.7%	1.000	< 0.0001
Atrial fibrillation	10.3%	13.1%	0.002	0.085	10.6%	10.3%	0.803	0.009
Cerebrovascular disease	17.8%	9.7%	< 0.0001	0.225	16.4%	16.3%	0.957	0.002
Chronic pulmonary disease	11.7%	12.6%	0.306	0.027	12.0%	12.3%	0.817	0.009
Coagulopathy	0.3%	0.4%	0.774	0.008	0.4%	0.2%	0.480	0.027
Dementia	3.1%	4.2%	0.038	0.057	3.3%	3.4%	0.912	0.004
Depression	15.8%	9.0%	< 0.0001	0.207	14.7%	14.6%	0.956	0.002
Heart failure	33.1%	15.9%	< 0.0001	0.408	30.5%	29.4%	0.501	0.023
Hypertension	72.5%	44.0%	< 0.0001	0.604	70.6%	69.9%	0.612	0.016
Liver disease	2.9%	0.9%	< 0.0001	0.145	2.5%	2.7%	0.714	0.013
Malignancy	10.4%	11.7%	0.113	0.042	10.7%	11.2%	0.664	0.016
Paralysis	1.1%	0.4%	< 0.0001	0.084	1.1%	1.1%	0.853	0.007
Peripheral vascular disease	26.8%	5.5%	< 0.0001	0.605	22.9%	23.1%	0.875	0.005
Prior CABG	7.9%	2.7%	< 0.0001	0.233	6.9%	6.6%	0.762	0.011
Prior MI	21.1%	12.4%	< 0.0001	0.234	19.7%	19.8%	0.921	0.004
Psychotic disorder	3.3%	2.9%	0.357	0.023	3.3%	3.5%	0.753	0.012
Rheumatic disease	6.7%	6.1%	0.373	0.023	6.9%	6.6%	0.762	0.011
Renal failure	24.3%	1.9%	< 0.0001	0.703	18.8%	19.7%	0.387	0.022
Valvular disease	5.6%	5.2%	0.411	0.021	5.4%	6.3%	0.324	0.036
ST-elevation MI	30.2%	40.0%	< 0.0001	0.205	31.3%	32.7%	0.363	0.031
Anterior*	53.3%	46.7%	0.084	0.081	53.0%	50.7%	0.489	0.046
Revascularization	58.4%	62.7%	0.001	0.089	59.9%	60.7%	0.621	0.018
PCI	48.3%	56.1%	< 0.0001	0.156	49.8%	50.0%	0.934	0.003
CABG	11.1%	7.3%	< 0.0001	0.130	11.1%	11.3%	0.852	0.007
Post-MI medication								
ACEi or ARB	70.0%	68.0%	0.089	0.045	71.1%	71.5%	0.822	0.008
Aldosterone antagonist	5.4%	3.3%	< 0.0001	0.100	5.6%	6.2%	0.519	0.024
Antiarrhythmic	1.2%	1.2%	0.871	0.004	1.1%	1.1%	1.000	< 0.0001
Beta-blocker	84.6%	84.5%	0.888	0.004	84.9%	86.5%	0.201	0.046
Oral anticoagulant	11.7%	13.4%	0.058	0.051	12.0%	10.6%	0.224	0.045
P2Y_12_ inhibitor	68.4%	70.0%	0.176	0.035	69.2%	70.3%	0.485	0.025
Statin	81.8%	84.2%	0.012	0.064	83.3%	83.9%	0.264	0.042
University Hospital	55.5%	51.0%	0.001	0.099	55.0%	54.0%	0.547	0.022
Year of index MI			< 0.0001	0.173			0.801	0.007

Features of all patients and propensity score-matched cohort. *CABG* coronary artery bypass grafting surgery, *MI* myocardial infarction, *PCI* percutaneous coronary intervention, *SMD* standardized mean difference. *Of ST elevation MI patients

We matched type 1 diabetes patients 1:1 with control patients without diabetes using the optimal matching method without replacement with a caliper set at 0.2 times the standard deviation of the estimated propensity score. Multivariable regression models included the same variables as in the propensity scores (except for the year of index MI). The extent of potential unmeasured confounding was estimated by calculating the E-value [16]. Results are given as the mean, percentage, sub-distribution hazard ratio (sHR), or odds ratio (OR) with 95% CI, IQR, or ± SD. Statistical significance was inferred at a p value of < 0.05. Analyses were performed using SAS version 9.4 (SAS Institute Inc., Cary, NC, USA).

## Results

The study population included 1,508 patients with type 1 diabetes and 62,785 patients without any type of diabetes. In the original cohort, patients with type 1 diabetes were younger, more often female, and had a higher cardiac, vascular, and renal co-morbidity burden than patients without diabetes (Table 1). They also presented with non-ST-elevation MI more often compared to patients without diabetes. Revascularization was less frequently performed for patients with type 1 diabetes, but treatment with CABG was more common in type 1 diabetes. Baseline differences were balanced with propensity score matching resulting in 1,401 patients with type 1 diabetes and 1,401 patients without diabetes (Table 1).

### Major adverse cardiovascular event

**Fig. 1:**
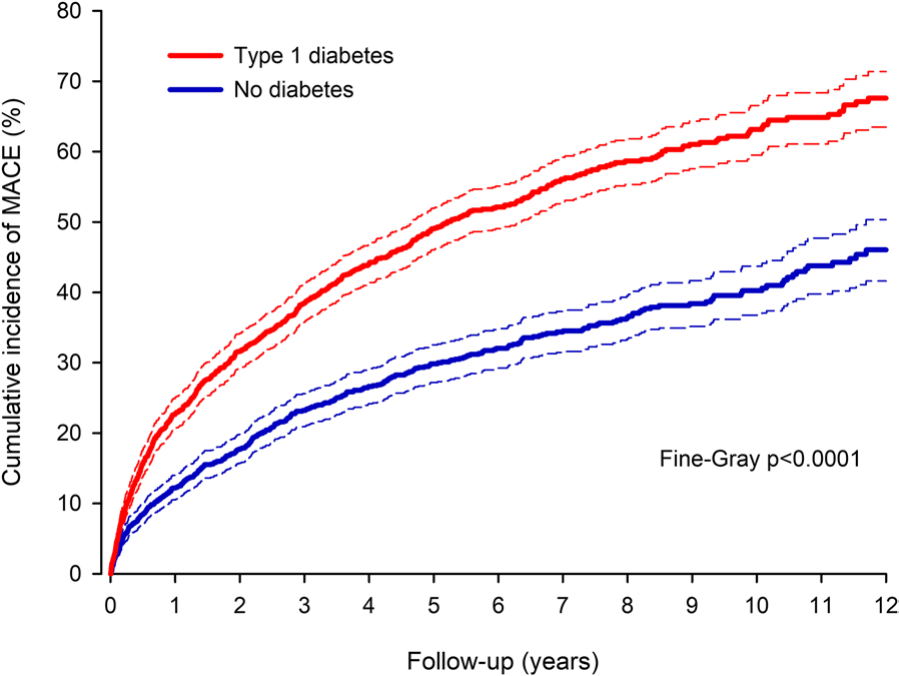
Cumulative incidence of major adverse cardiovascular events after myocardial infarction. Cumulative incidence of major adverse cardiovascular events (MACE) after myocardial infarction in patients with type 1 diabetes and in matched control patients without any type of diabetes. Dashed lines represent 95% confidence intervals. Number of events and patients at risk are presented in Additional file 1: Table S3

The numbers of outcome events are presented in Additional file 1: Table S1. The occurrence of a MACE was higher in patients with type 1 diabetes compared to non-diabetic controls after MI in the total study population (Additional file 1: Fig. S2) and in the matched population (Fig. 1). In the total study cohort, the 12 year cumulative incidence of MACE was 68.4% in the type 1 diabetes group and 45.3% in the group without diabetes (multivariable adjusted sHR 2.22; CI 2.02–2.43; p < 0.0001). In the matched cohort, the one-year cumulative incidence of MACE was 22.8% in the type 1 diabetes group versus 12.2% in the group without diabetes (p < 0.0001) and 49.0% versus 29.8%, respectively, at five years (p < 0.0001). During the 12-year follow-up, the cumulative incidence of MACE was 67.6% in type 1 diabetes patients versus 46.0% in matched non-diabetic patients (sHR 1.94; CI 1.74–2.17; p < 0.0001). The E-value was 3.29 (CI 2.87–3.76).

### Secondary outcomes

**Fig. 2:**
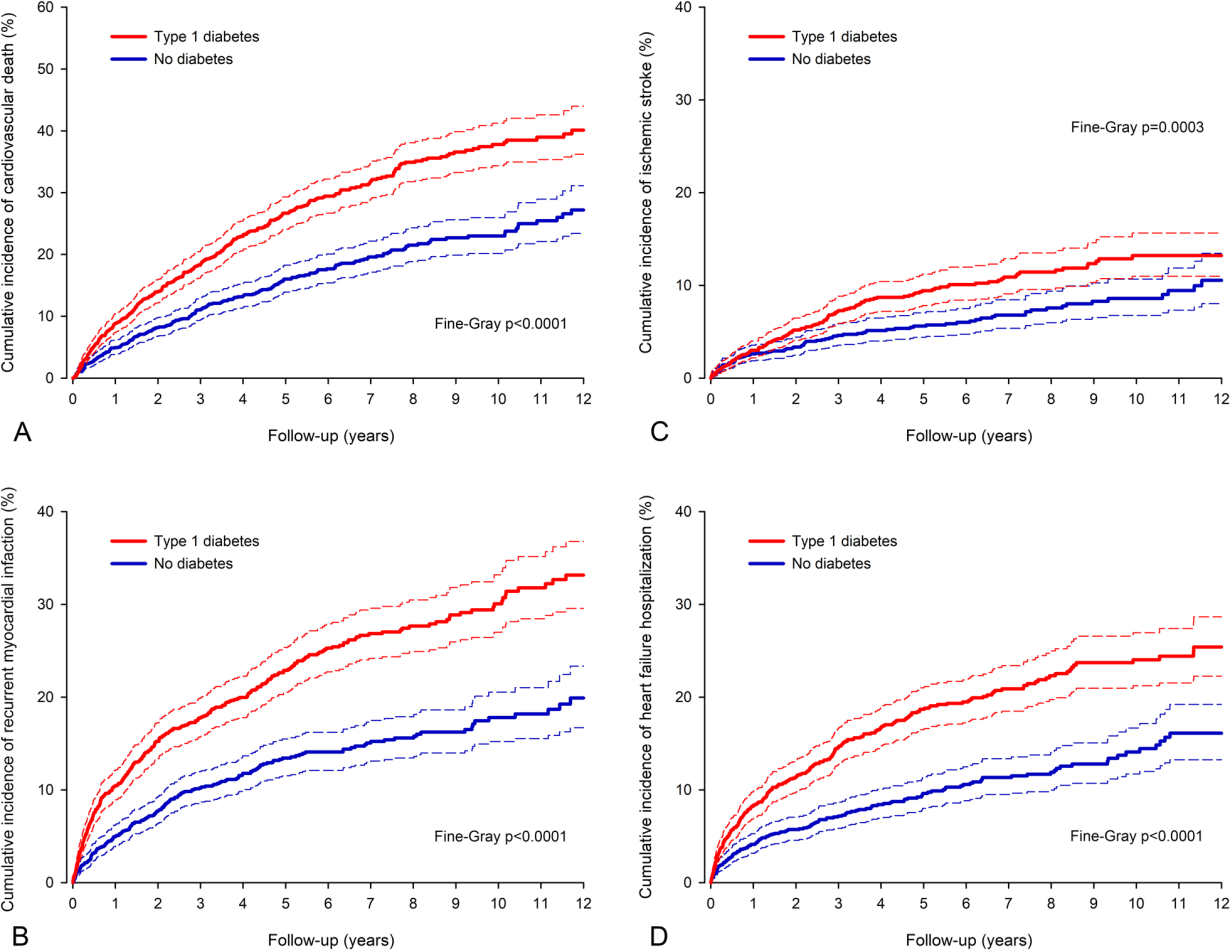
Cumulative incidence of secondary outcomes after myocardial infarction. Cumulative incidence of cardiovascular death (A) and hospitalization for recurrent myocardial infarction (B), ischemic stroke (C), and heart failure (D) after index myocardial infarction in patients with type 1 diabetes and in matched control patients without any type of diabetes. Please note the differences in y-axis. Dashed lines represent 95% confidence intervals

The probabilities of cardiovascular death and hospitalization for recurrent MI, ischemic stroke, and heart failure (Fig. 2) were all higher in patients with type 1 diabetes compared to matched control patients without diabetes. The cumulative incidence of cardiovascular death after MI was 8.9% in type 1 diabetes patients versus 5.0% in matched non-diabetic patients at one-year follow-up (p < 0.0001) and 40.1% versus 27.2%, respectively, during the complete 12-year follow-up (sHR 1.81; CI 1.56–2.09; p < 0.0001).

The cumulative incidence of a recurrent MI was 12.2% in the type 1 diabetes group versus 5.0% in the matched control group at one-year follow-up (p < 0.0001) and 36.8% versus 19.9% within the 12-year follow-up period (sHR 1.91; CI 1.64–2.23; p < 0.0001). Correspondingly, the cumulative incidence of ischemic stroke was 3.0% versus 2.6% at one year (p = 0.326) and 13.5% versus 10.6% at 12 years (sHR 1.50; CI 1.21–1.87; p = 0.0003). The cumulative incidence of heart failure hospitalization was 8.3% in type 1 diabetes patients versus 4.1% at one year (p < 0.0001) and 25.4% versus 16.1% during the complete follow-up (sHR 1.98; CI 1.67–2.35; p < 0.0001). Results of multivariable analyses in the total study cohort were comparable to results of the matched cohort (Additional file 1: Table S2).

### Subgroups

**Fig. 3:**
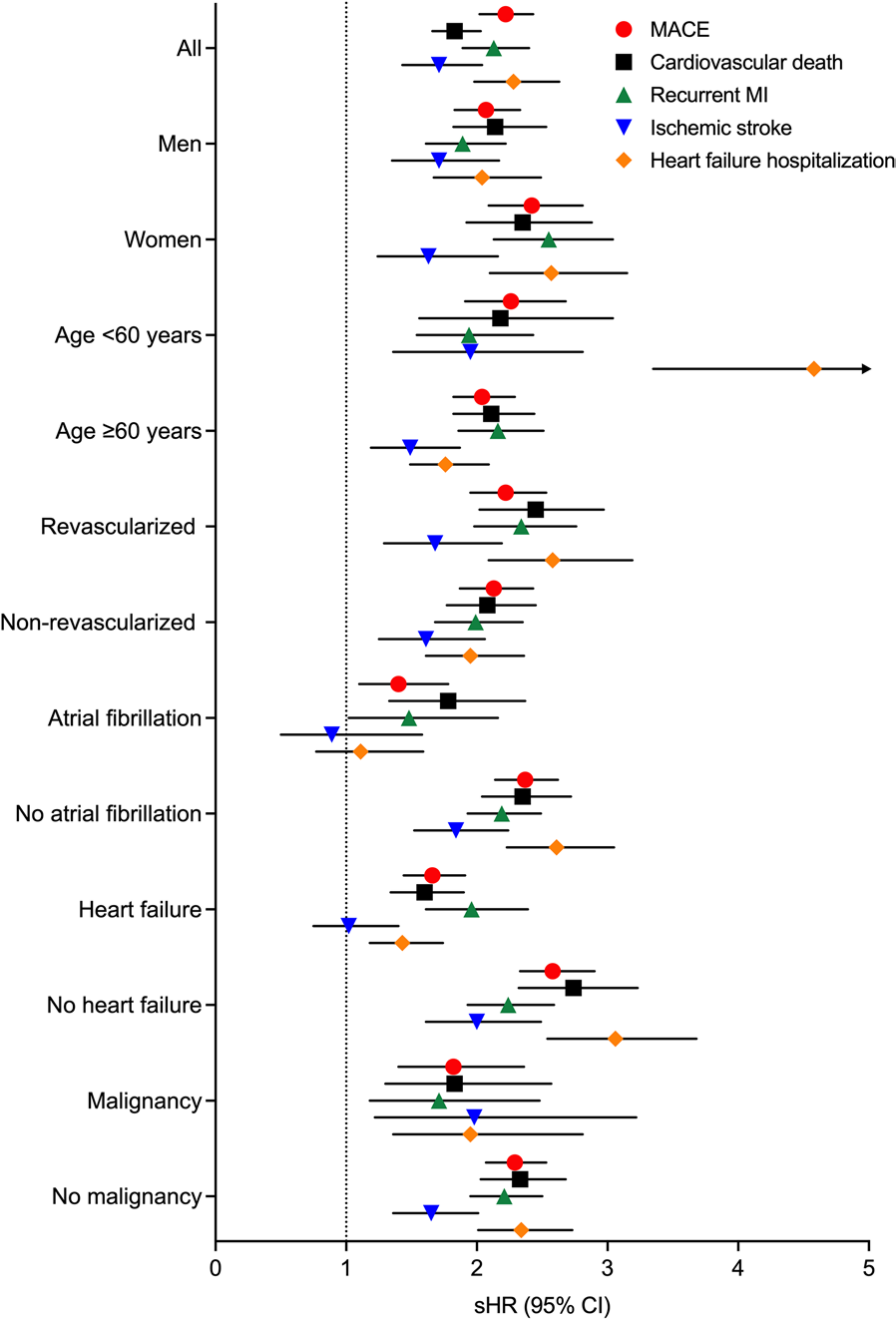
Multivariable-adjusted subdistribution hazard ratios for 12-year outcomes comparing patients with type 1 diabetes vs. patients without diabetes in subgroups. Patient subgroups are listed on y-axis. Shapes represent sHRs and whiskers 95% confidence intervals (CIs). Abbreviations: MACE, major adverse cardiovascular event; MI, myocardial infarction; sHR, subdistribution hazard ratio

The hazard of MACE was higher in type 1 diabetes patients than in patients without diabetes in subgroups of men and women, patients aged < 60 and ≥ 60 years, revascularized and non-revascularized patients, and patients with and without atrial fibrillation, heart failure or malignancy at baseline (Fig. 3). Results relative to cardiovascular death and recurrent MI were also consistent in these subgroups. Type 1 diabetes-related excess hazard of ischemic stroke was also apparent in various subgroups, with the exceptions of patients with baseline atrial fibrillation and baseline heart failure. Likewise, type 1 diabetes-related excess hazard of heart failure hospitalization was consistent in the subgroup analyses, with the exception of patients with atrial fibrillation (Fig. 3).

### Usage of cardiovascular medication

Secondary preventive ACE inhibitors/angiotensin receptor blockers (ARBs) and P2Y_12_-inhibitors were more frequently used in patients with type 1 diabetes compared to propensity score-matched patients without diabetes (Table 2). Statins, beta-blockers, and oral anticoagulants were used by a similar proportion in the matched study groups (Table 2).

**Table 2:** Secondary preventive cardiovascular prescription medication after myocardial infarction in patients with type 1 diabetes and matched controls without any type of diabetes

	Type 1 Diabetes	No Diabetes		
	n = 1403	n = 1403	OR (95% CI)	P value
ACEi or ARB	72.0%	66.2%	1.32 (1.12–1.56)	0.001
Aldosterone antagonist	5.7%	4.6%	1.24 (0.89–1.74)	0.204
Beta-blocker	85.2%	82.8%	1.11 (0.90–1.27)	0.318
Oral anticoagulant	11.7%	13.7%	0.82 (0.65–1.04)	0.101
P2Y_12_ inhibitor	69.4%	64.9%	1.25 (1.06–1.48)	0.008
Statin	81.8%	82.7%	0.94 (0.77–1.15)	0.540

Controls were matched with a separate propensity score including sex, age, all co-morbidities listed in Table 1, revascularization by PCI or CABG, ST-elevation, year of index MI, and treating hospital (university versus non-university)

*ACEi* angiotensin-converting-enzyme inhibitor, *ADP* adenosine diphosphate, *ARB* angiotensin receptor blocker, *OR* odds ratio, *CI* confidence interval

## Discussion

### Main findings in relation to previous literature

The main finding of this nationwide registry study was that the long-term hazard of major adverse cardiovascular events following an MI was notably higher among patients with type 1 diabetes compared to patients without diabetes but with otherwise similar baseline features including comorbidities, age distribution, and rates of revascularization. The results were consistent regarding the hazard of cardiovascular death, recurrent MI, ischemic stroke, and heart failure hospitalization. The 12-year cumulative incidences of cardiovascular events after MI in patients with type 1 diabetes were alarmingly high: 68% for MACE, 40% for cardiovascular death, 37% for recurrent MI, 14% for ischemic stroke, and 25% for heart failure hospitalization.

Our previous study demonstrated that patients with type 1 diabetes have higher short-term (30-day and 1-year) case fatality rates after MI compared to patients without diabetes while sharing otherwise similar baseline features [12]. Patients with type 1 diabetes undergoing PCI seemed to be at a particularly high risk of adverse in-hospital outcomes including MACE, mortality, and major bleeding [17]. The present study extended these findings by showing that long-term (median follow-up 3.9 years) cardiovascular prognosis after MI in patients with type 1 diabetes compared to non-diabetic controls was substantially poorer. Similar findings of impaired long-term outcomes after MI, i.e., higher long-term mortality rates and higher occurrence of recurrent MIs and heart failure, have been revealed in cohorts with type 2 diabetes or with diabetes of unspecified type but, to the best of our knowledge, not in cohorts with type 1 diabetes [6, 7, 18, 19]. Many studies have also disclosed poorer outcomes among insulin-treated compared to non-insulin treated patients with diabetes after MI or PCI [10, 11, 20, 21] but have not specifically examined patients with type 1 diabetes.

### Risk factors for CVD in type 1 diabetes

Reasons for the increased CVD risk in type 1 diabetes are multifaceted and include both traditional and diabetes-specific risk factors. Poor glycemic control and presence of microvascular disease (diabetic nephropathy, diabetic retinopathy or cardiac autonomic neuropathy) are associated with increased risk of CVD in type 1 diabetes [22–24]. CVD risk is more pronounced among patients with earlier onset of type 1 diabetes compared to those with later onset [22]. Endothelial dysfunction, oxidative stress, vascular inflammation, and immune dysfunction may play a role as underlying mechanisms [22]. Furthermore, metabolic syndrome and central obesity are associated with an increased risk of macrovascular complications and heart failure hospitalizations, respectively, in adults with type 1 diabetes [25, 26]. Physical activity levels and cardiorespiratory fitness may be poorer among young patients with type 1 diabetes compared to apparently healthy peers [27].

Glycemic control in type 1 diabetes is a key determinant of not only the risk of first but also subsequent cardiovascular events [28]. In the setting of CABG among patients with type 1 diabetes, poor glycemic control is associated with an increased long-term risk of cardiovascular events and death [29]. It may be hypothesized that the same applies to the setting of MI as well.

### Role of revascularization status and the extent of coronary artery disease

Diabetes has consistently been shown to be an important risk factor for poor outcomes after PCI, even in the era of drug-eluting stents [30]. Importantly, outcomes after PCI with drug-eluting stents may be poorer in patients with insulin-dependent diabetes compared to those with non-insulin-dependent diabetes [31]. In our study, approximately 60% of propensity score-matched patients with type 1 diabetes and controls without diabetes were revascularized (50% underwent PCI and 11% underwent CABG). Long-term cardiovascular outcomes after MI were poorer among patients with type 1 diabetes than in controls, regardless of whether they were revascularized.

Patients with type 1 diabetes with an indication of coronary angiography are characterized by more severe and more extensive coronary atherosclerosis than controls without diabetes [32], which may be one reason for our findings. According to a Swedish registry study of more than 2,700 patients with type 1 diabetes undergoing coronary angiography for various reasons (acute coronary syndrome (ACS) in 48%), mortality was increased by the number of affected coronary arteries [33]. In that study, the majority of patients with type 1 diabetes and ACS had either multi-vessel or left main disease, whereas 23% of patients with non-ST-elevation ACS and 40% of ST-elevation MI had one-vessel disease. Future studies may explore how much the differences in the extent of coronary atherosclerosis contribute to outcome differences between type 1 diabetes patients and controls after MI.

### Risk of heart failure hospitalizations after MI

The risk of heart failure is increased in both type 2 and type 1 diabetes patients [34]. Our observation of an increased probability of heart failure hospitalizations after MI in patients with type 1 diabetes was in line with a Swedish nationwide registry study in which the presence of diabetes (of unspecified type) increased the risk of heart failure after first MI (adjusted HR 1.52; 95% CI 1.44–1.61) [7].

Varying myocardial pathologies can occur in patients with diabetes, which may, in theory, contribute to the higher risk of new cardiovascular events, especially heart failure, among patients with type 1 diabetes experiencing an MI. Diabetic cardiomyopathy has been less extensively studied in type 1 diabetes compared to type 2 diabetes and has both shared and distinct characteristics in these two diabetes subtypes [35, 36]. In type 2 diabetes, diabetic cardiomyopathy often presents with preserved ejection fraction. In type 1 diabetes, systolic dysfunction and classic heart failure symptoms are more typical and occur earlier in the course of diabetic cardiomyopathy [36]. Patients with type 1 diabetes and type 2 diabetes both often present with abnormal diastolic function compared to measurements in healthy controls, although diastolic dysfunction is more common in patients with type 2 diabetes compared to patients with type 1 diabetes [37]. The diabetic myocardium may be more prone to acute ischemia–reperfusion injury compared to a non-diabetic setting [38]. Patients with type 1 diabetes, especially those with poor glycemic control, may develop cardiac autoantibodies that are associated with future CV events [39]. It has been suggested that a distinct post-MI cardiac autoimmune syndrome may exist in type 1 diabetes [40].

### Use of secondary preventive medications

After a separate propensity score matching, we observed a few noteworthy differences in the use of evidence-based secondary preventive medications between patients with type 1 diabetes and control subjects. Patients with type 1 diabetes were more likely using ACE inhibitors and/or ARBs as well as P2Y_12_ inhibitors. The first difference may be explained by ACE inhibitors and ARBs being the preferred agents in the management of hypertension in patients with diabetes and because of their beneficial effects on albuminuria in diabetic nephropathy. Of note, the usage rates of both ACE inhibitors / ARBs and P2Y_12_ inhibitors were low in both study groups indicating room for improvement. Although the presence of diabetes may be associated with lower statin adherence after MI according to previous studies [41], frequency of statin use did not differ significantly between patients with type 1 diabetes and controls in our study. Underutilization of evidence-based treatments, including statins, has been suggested to explain worse outcomes after MI among patients with diabetes [42], but our results do not support this theory in type 1 diabetes. However, only roughly 80% of MI patients used statins during the three-month period after the index MI (as measured by dispensed medications), leaving potential for improvement in both patients with and without type 1 diabetes.

### Future directions for research and clinical practice

Our results may prompt health care professionals and patients with type 1 diabetes to strive for better cardiovascular risk factor control after MI. This includes smoking cessation, control of blood pressure and cholesterol levels, and glycemic control, by means of both lifestyle modifications and pharmacological therapies. In type 2 diabetes, physicians have the opportunity to initiate cardioprotective glucose-lowering agents, such as glucagon-like peptide-1 receptor agonists or sodium-glucose cotransporter-2 inhibitors, after MI to reduce subsequent cardiovascular events [43]. Currently, none of these drugs has an indication for use in type 1 diabetes, in which the risk of ketoacidosis remains a concern. Therefore, it would be important to conduct studies investigating these medications in patients with type 1 diabetes, especially regarding their effects on cardiovascular risk.

### Strengths and limitations

Major strengths of the current study include the use of nationwide registry data during a long follow-up and validation for diagnoses for fatal and non-fatal CHD events, heart failure, and stroke [44–46]. However, the available coding does not allow for reliable identification of in-hospital or post-operative MIs, although MI was required to be the primary discharge diagnosis. We used a multi-registry linkage approach for identification of type 1 diabetes. Keeping in mind the possibility of erroneous recording of diagnostic codes, some patients with type 2 diabetes (or some other type of diabetes) who received no other treatment than insulin may have been falsely identified as patients with type 1 diabetes.

Although we accounted for several comorbidities and baseline features in our analyses, data on several important factors, such as ECG, coronary angiography findings, and extent of revascularization, laboratory results such as HbA1c and glomerular filtration rate, and smoking were unavailable to us and may have caused unmeasured confounding. However, the analysis of E-value [16] indicated that an unmeasured confounder would need to have an association of at least 3.3 on the risk ratio scale with both the presence of type 1 diabetes and occurrence of MACE to be able to fully account for the observed difference in MACE between patients with type 1 diabetes and matched control subjects. To study the usage of secondary preventive medications, the follow-up started 90 days post MI and early MACE events were therefore not captured. Furthermore, low-dose aspirin can be purchased without prescription from pharmacies in Finland, and therefore we could not identify its use reliably from registry data.

## Conclusions

In this nationwide registry study, we observed higher long-term rates of cardiovascular events after MI in patients with type 1 diabetes compared to patients without diabetes. When planning secondary prevention of CVD events in patients with a previous MI, type 1 diabetes should be regarded as a significant risk factor for recurrence. Informing patients with type 1 diabetes about this excess risk after MI may motivate them to pursue lifestyle modifications and medication adherence.

## Supplementary Information

**Additional file 1: Figure S1.** Study flow-chart. **Figure S2.** Cumulative incidence of major adverse cardiovascular event (MACE) after myocardial infarction in patients with type 1 diabetes and in unmatched control patients without any type of diabetes. **Table S1.** Number of outcome events during 12-year follow-up. **Table S2.** Cumulative incidences of outcomes in the total study cohort during the 12-year follow-up. **Table S3. **Number of major adverse cardiovascular events (MACE), competing events (non-cardiovascular death), censored patients, and patients at risk during follow-up of patients with type 1 diabetes and of matched control patients without any type of diabetes after myocardial infarction.

## Data Availability

Because of the sensitive nature of the data collected for this study, requests to access the data set from qualified researchers trained in human subject confidentiality protocols may be sent to the Findata at http://findata.fi/en.

## Abbreviations

ACE: Angiotensin converting enzyme
ACS: Acute coronary syndrome
ARB: Angiotensin receptor blocker
CABG: Coronary artery bypass grafting
CHD: Coronary heart disease
CI: Confidence interval
CVD: Cardiovascular disease
CRHC: The Care Register for Health Care in Finland
GDPR: General Data Protection Regulation
ICD-10: International Classification of Diseases 10th Revision
IQR: Interquartile range
MACE: Major adverse cardiovascular event
MI: Myocardial infarction
OR: Odds ratio
PCI: Percutaneous coronary intervetion
sHR: Subdistribution hazard ratio
SMD: Standardized mean difference
