# Supplementary material for: Long-term cardiovascular prognosis of patients with type 1 diabetes after myocardial infarction
Source: Cardiovasc Diabetol. 2022 Sep 6;21:177. doi: 10.1186/s12933-022-01608-3 (PMC9450422; doi:10.1186/s12933-022-01608-3)
Supplement: Supplementary file 1 — Additional file 1: Figure S1. Study flow-chart. Figure S2. Cumulative incidence of major adverse cardiovascular event (MACE) after myocardial infarction in patients with type 1 diabetes and in unmatched control patients without any type of diabetes. Table S1. Number of outcome events during 12-year follow-up. Table S2. Cumulative incidences of outcomes in the total study cohort during the 12-year follow-up. Table S3. Number of major adverse cardiovascular events (MACE), competing events (non-cardiovascular death), censored patients, and patients at risk during follow-up of patients with type 1 diabetes and of matched control patients without any type of diabetes after myocardial infarction. [file 12933_2022_1608_MOESM1_ESM.pdf]

Additional file 1

<u>Additional Methods</u>

Outcome definitions:

- Major adverse cardiovascular event: Cardiovascular death, recurrent myocardial infarction hospitalization, ischemic stroke hospitalization, or heart failure hospitalization
- Cardiovascular death: ICD-10 code I* as the underlying cause of death
- Recurrent myocardial infarction hospitalization: ICD-10 code I21* or I22* as the primary diagnosis in hospital admission
- Ischemic stroke hospitalization: ICD-10 code I63* as any diagnosis in hospital admission
- Heart failure hospitalization: ICD-10 code I50* as primary diagnosis in hospital admission

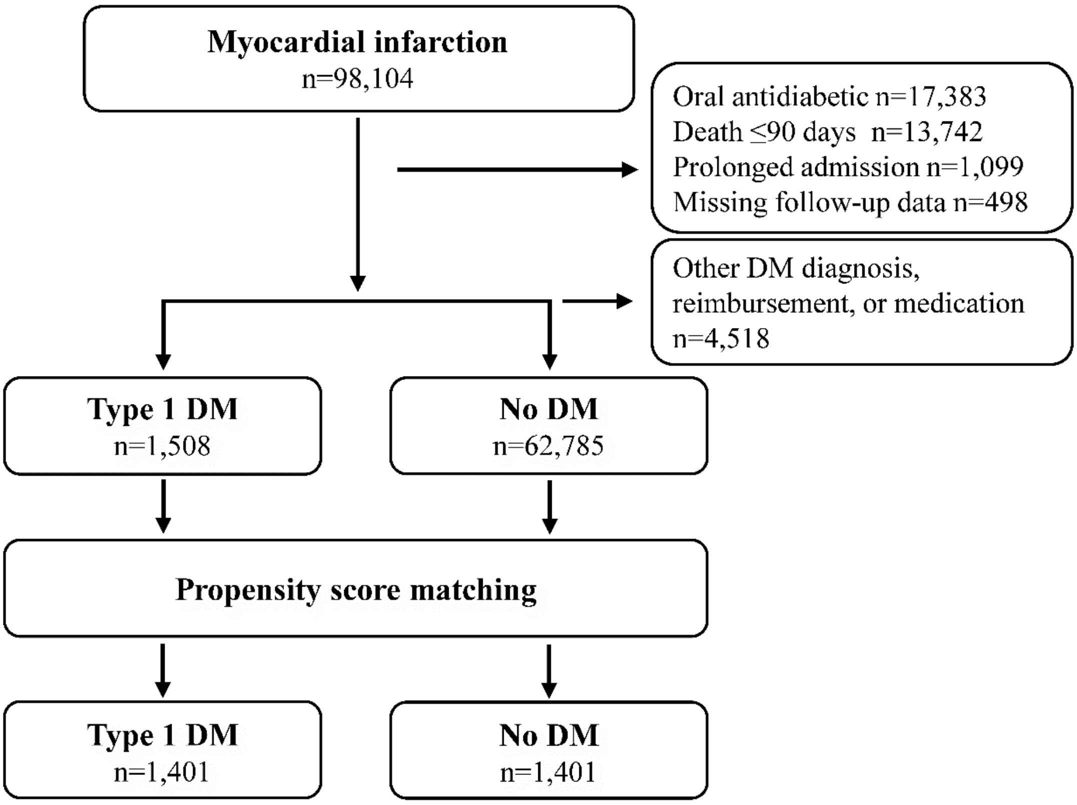

Figure S1. Study flow-chart.

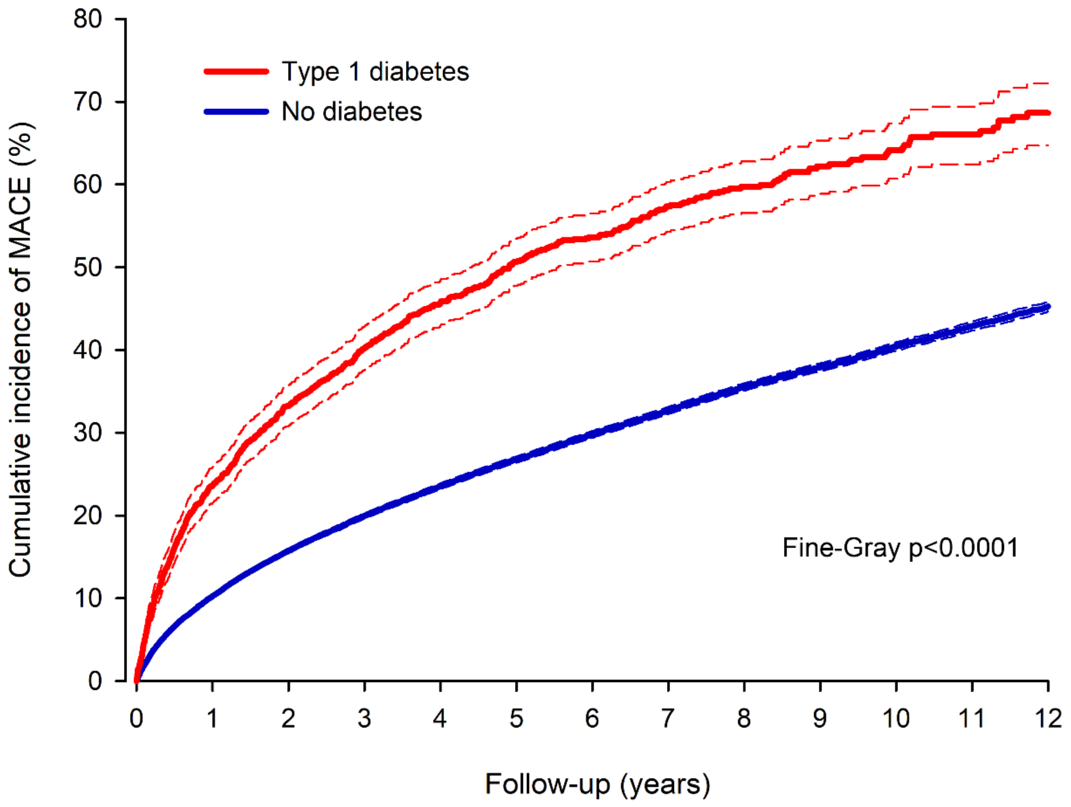

Figure S2. Cumulative incidence of major adverse cardiovascular event (MACE) after myocardial infarction in patients with type 1 diabetes and in unmatched control patients without any type of diabetes. Dashed lines represent 95% confidence intervals.

	All patients		Matched patients	
**Outcome event**	**T1DM**	**No DM**	**T1DM**	**No DM**
MACE	754	18,955	683	427
Cardiovascular death	411	10,943	378	233
Recurrent myocardial infarction	361	14,350	327	185
Ischemic stroke	146	3,944	131	88
Heart failure hospitalization	279	6,088	257	143

Table S1. Number of outcome events during 12-year follow-up.

	Cumulative incidence		Univariable		Multivariable	
**Outcome**	**Type 1 Diabetes**	**No Diabetes**	**sHR (95%CI)**	***P*-value**	**sHR (95%CI)**	***P*-value**
**MACE**	68.4%	45.3%	2.18 (2.02-2.35)	<0.0001	2.22 (2.02-2.43)	<0.0001
**Cardiovascular death**	40.4%	28.3%	1.83 (1.66-2.03)	<0.0001	1.83 (1.66-2.03)	<0.0001
**Recurrent myocardial infarction**	33.6%	16.8%	2.40 (2.16-2.67)	<0.0001	2.13 (1.89-2.40)	<0.0001
**Ischemic stroke**	13.5%	10.0%	1.65 (1.40-1.95)	<0.0001	1.71 (1.43-2.04)	<0.0001
**Heart failure hospitalization**	25.5%	14.8%	2.15 (1.90-2.42)	<0.0001	2.28 (1.98-2.63)	<0.0001

Table S2. Cumulative incidences of outcomes in the total study cohort during the 12-year follow-up. Results of unadjusted and multivariable adjusted Fine-Gray models (see methods for details). sHR = subdistribution hazard ratio.

	**Type 1 diabetes / No diabetes**			
**Follow-up (years)**	**MACE**	**Competing event**	**Censored**	**At risk**
**0**	0 / 0	0 / 0	0 / 0	1401 / 1401
**2**	423 / 236	53 / 41	170 / 209	755 / 915
**4**	552 / 330	79 / 67	321 / 390	449 / 614
**6**	617 / 373	93 / 78	415 / 529	276 / 421
**8**	655 / 399	99 / 85	502 / 649	145 / 268
**10**	672 / 414	103 / 93	543 / 756	83 / 138
**12**	683 / 427	106 / 94	612 / 880	35 / 48

Table S3. Number of major adverse cardiovascular events (MACE), competing events (non-cardiovascular death), censored patients, and patients at risk during follow-up of patients with type 1 diabetes and of matched control patients without any type of diabetes after myocardial infarction. Please refer to Figure 1.
